# Zinc(II) niflumato complex effects on MMP activity and gene expression in human endometrial cell lines

**DOI:** 10.1038/s41598-021-98512-9

**Published:** 2021-09-27

**Authors:** Miroslava Rabajdová, Ivana Špaková, Zuzana Klepcová, Lukáš Smolko, Michaela Abrahamovská, Peter Urdzík, Mária Mareková

**Affiliations:** 1 Department of Medical and Clinical Biochemistry, Faculty of Medicine, Pavol Jozef Šafárik University, Trieda SNP 1, 040 11 Košice, Slovakia; 2 Department of Gynaecology and Obstetrics, Faculty of Medicine, Pavol Jozef Šafárik University, Košice, Slovakia

**Keywords:** Biochemistry, Drug discovery, Molecular biology, Diseases, Medical research, Molecular medicine, Chemistry

## Abstract

Endometriosis is a chronic inflammatory disease which increasingly affects young women under 35 years of age and leads to subfertility even infertility. Analysis of the cytotoxic effect of zinc(II) niflumato complex with neocuproine ([Zn(*neo*)(*nif*)_2_] or Zn-Nif) on immortalized human endometriotic cell line (12Z) and on control immortalized human endometrial stromal cell line (hTERT) was performed using xCELLigence technology for approximately 72 h following the treatment with Zn-Nif as well as cell viability Trypan Blue Assay. 12Z cell line proliferated more slowly compared to unaffected cells, whereas hTERT cells did not show similar behavior after treatment. The complex probably reduces the effect of pro-inflammatory pathways due to the effect of NSAID, while presence of zinc might reduce the level of ROS and regulate ER2 levels and MMP activity. The observed effects and high selectivity for rapidly proliferating cells with increased inflammatory activity suggest a good prognosis of successful decrease of endometriosis stage with this complex.

## Introduction

Thickness of cylindrical endometrial epithelium changes cyclically due to the menstrual and ovarian cycles^1^ controlled by periodically produced gonadotropins (GnRH) in the hypothalamus such as FSH (follicle stimulating hormone) and LH (luteinizing hormone)^2^. GnRHs are influenced by feedback levels of estrogens (mainly estradiol) and progesterones^3^. Endometriosis is defined as the presence of a endometrium-like tissue (glands, endometrial epithelial and stromal cells forming benign implants) in an area not typical for its occurrence—outside the uterine cavity^4^ as a consequence of hormonal imbalance associated with immune deficiency and inflammatory processes^5^. Endometriosis tissue behaves similarly to a healthy endometrium, but is not excreted from the body by menstruation, decomposes, forms adhesions and is subject to develop vast inflammation^6^. It is often accompanied by pain in the pelvic floor, especially during the menstrual period, during and after sexual intercourse^6^ as scarring of the uterine cavity and the formation of adhesions occurs^7^. It also presents an increased risk of ovarian cyst formation (ovarian endometriosis), which causes reduced fertility and may even result in ovarian cancer^8^.

This chronic disease is a particularly common condition and occurs in up to 10% of women of childbearing age (presentation of the disease before the first period or after menopause is little known)^9,10^. 2–50% of affected women do not show the symptoms of disease (silent endometriosis), 40–60% of women suffer from painful menstruation and 20–30% of women are unable to conceive (subfertile to infertile)^6^.

Unfortunately, the etiopathogenesis of the endometriosis remains unclear and there is no indisputable theory of its origin. The one of the most accepted cause of endometriosis implants is Sampson's theory of transplantation, in which viable endometrial epithelial and stromal cells, enters the peritoneal cavity through retrograde menstruation^7^, resulting in increased concentrations of iron accumulated in macrophages and mediates oxidative stress^11^.

Based on the localization, we can divide endometriotic tissues into three groups: (1) eutopic endometrium (endometrium in the uterine cavity); (2) ectopic endometrium (endometrium in ectopic cells in the peritoneum and abdominal cortes); (3) endometriosis lesions^12,13^. It distinguishes three forms of pelvic endometriosis: (1) peritoneal endometriosis, (2) ovarian endometriosis, (3) deeply infiltrated endometrial lesions^7^. Endometriosis rarely occurs extraperitoneally (as a result of deep infiltration) in the colon, kidneys, liver, pancreas or lungs^7,14^.

### Biogenesis of endometriosis genitalis interna–endometriosis corpus uteri

Refluxed endometrial or endometrial-like cells in the peritoneal cavity attach to the underlying mesothelium, form an ectopic lesion, secrete chemokines, and stimulate immune cell infiltration through tumour necrosis factor alpha (TNFα)^15^. Further through the feed-forward loop produces pro-inflammatory cytokines and prostaglandins, while anti-inflammatory interleukins are suppressed, resulting in an inflammatory imbalance^16,17^.

**Figure 1:**
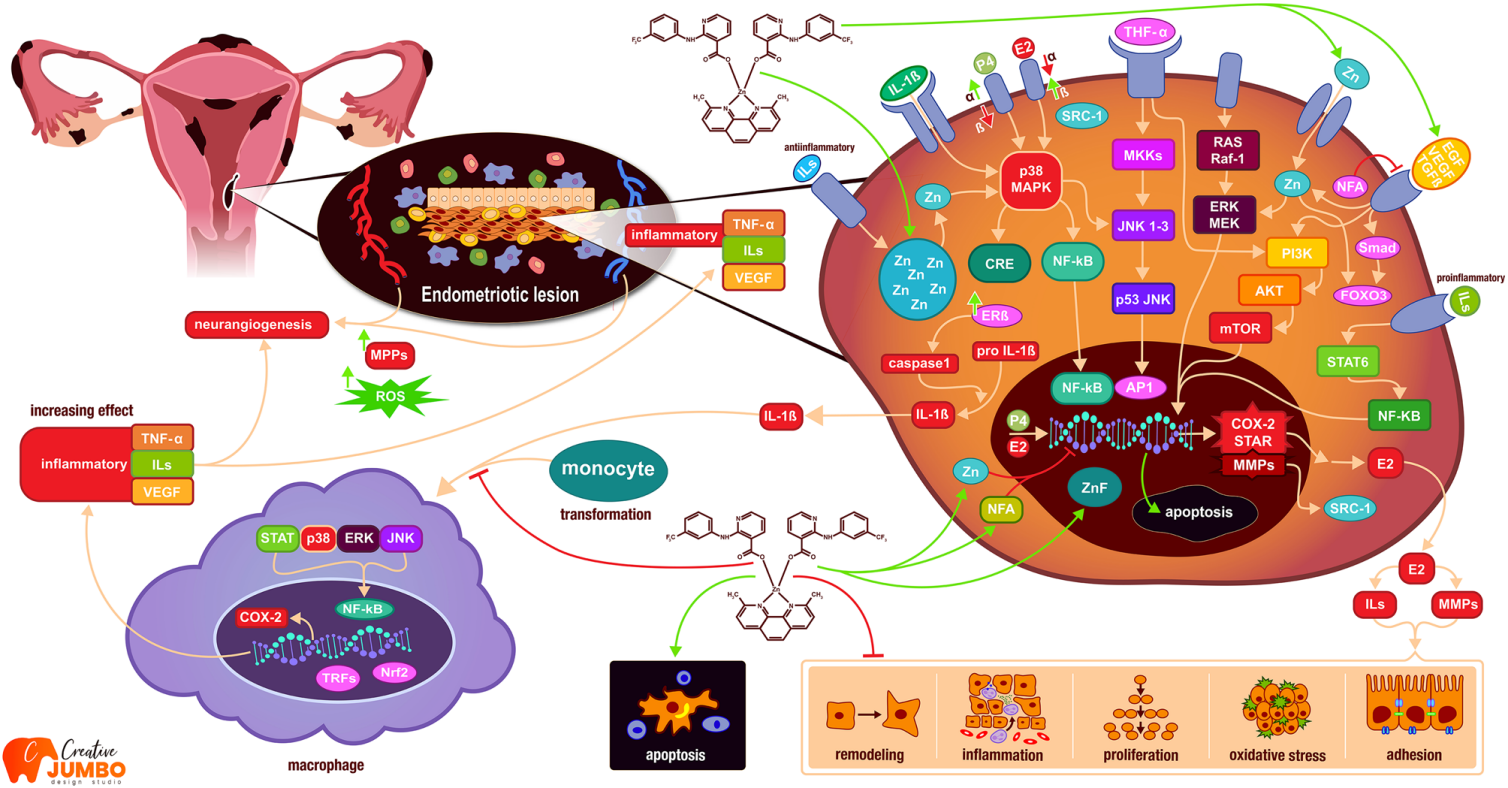
Mechanism of action of Zn-Nif complex on endometriotic cells.

Endometriotic lesions form a unique microenvironment that is able to resist apoptotic stimuli, induce kinase activity, overexpress telomerase, and survivin which are physiologically actively expressed in the ovaries and endometrium^18^. Endometriotic cells are characterized by epigenetic modifications, chromosomal anomalies, instability, and mutations in a specific DNA sequence, leading to deregulation of the pathways involved in endometriosis^7^. During neuroangiogenesis vascular endothelial growth factor (VEGF) plays a key regulatory role^17,19–21^. Regulation of the mitogen activated protein kinase (MAPK) pathway via epidermal growth factor (EGF), protease-activated receptors (PAR), and tyrosine kinases directly involves these signalling pathways in the extra- and intracellular regulation of endometrial pathogenesis^17^. MAPK signalling also affects pain hypersensitivity by activating cyclooxygenase (COX) which is likely to lead to pelvic floor pain^22^. COX levels are closely related to the local production of estrogen (E2) and progesterone (P4) which in imbalance boost the expression of COX and pro-inflammatory agents^22,23^. The resulting stress conditions support the pathological protease activity of matrix metalloproteinases (MMPs) whose activity depends on P4 and on the level of zinc (Zn)^24^. Endometriosis is responsible for subjective as well as objective changes (dysmenorrhea, dyspareunia, discretion, infertility, and irregular menstrual cycle) which together affect the patient's quality of life^25^. Influencing the activity of these kinases, proteinases, and hormone levels appears to be a potential target for treatment (Fig. 1).

### MAPK pathway in endometriosis

The ability of the endometriotic microenvironment to support endometriotic cells is transmitted through kinase signalling pathways. Deregulation of protein kinase leads to uncontrolled cell proliferation, inflammation, increased leaching of neurodegenerative mediators and steroid hormones, iron deposition, and subsequent oxidative stress contribute to the development of endometriosis^26^.

Mitogen-activated protein kinases (MAPK, MAP2K, MAP3K) are a family of protein kinases that control cell division by ERK1-8 (extracellular signal-regulated kinase), mediate the immune response, differentiation, and cell survival via p38α/β/γ/δ and regulate transcription by the JNK1-3 system (c-jun-N terminal kinase)^16,27^. Protein kinases together with phosphatases catalyse the transfer of a phosphate group to the side chain of a serine, threonine, or tyrosine specific protein^28^. Phosphorylation controls enzyme activity, interactions between individual proteins and molecules, their localization within the cell, and degradation by proteases^26^. MAPK-directed phosphorylation of specific serine and threonine residues in the target protein (protein kinases, phospholipases, and transcription factors) regulates processes from gene expression, mitosis, metabolism to apoptosis^17,29^.

The ERK/MAPK signalling cascade is stimulated by, for example, growth factors, cytokines, viral infections or carcinogens^30,31^. Upon activation of ERK1/2, a number of transcription factors and serine/threonine kinases are activated that direct cells to differentiate and survive^32^. Increased phosphorylation of ERK, p38 and JNK in eutopic endometrial epithelial cells correlates with long-acting phosphorylation in ectopic epithelial cells of endometriotic lesions, which are activated mainly by increased activity of TNFα, IL-1β (which induce IL-6/8), COX-2, TGFβ (transforming growth factor β) via Raf, PGE2 (along with increased activity of VEGF, MCPI) and ROS (H_2_O_2_)^16^.

The increase in migratory ability and invasiveness of ectopic endometriosis tissue via the ERK/MAPK signalling pathway correlates with overexpression of the angiogenic inflammatory factor TGF-β^33^. Cell migration is also supported by downregulation of cytokines that preserve vascular integrity and upregulation of MMPs (matrix metalloproteinases)^34^.

Dysregulation of the MAPK pathway in endometrial ectopic cells probably has a genetic basis and a positive family anamnesis is reported in 50% of patients^26^.

### The role of E2, P4 and MMP in inflammatory reactions due to COX-2 activation

Immune deficiency and persistent inflammatory environment stimulates immune system cells to non-physiological secretion of growth and angiogenic factors in endometriotic lesions^35^ promotes lesion implantation in the peritoneum, accelerates local inflammation, and grows in the ectopic endometrium^36^. The regulator of the immune system MIF (macrophage migration inhibitor factor) (in)directly affects endometrial tissue remodelling, proliferation, angiogenesis, and inflammatory responses^37^.

Pro-inflammatory and pathogenic stimuli at the transcriptional level (binding site in the COX gene promoter for NF-κB—nuclear factor-κB, CRE—cAMP-responsive element, MIF, NF-IL-6—nuclear factor for interleukin-6, HIF-1α (hypoxia-inducible factor 1α) and translational level (CHX, heavy metals—Cd, eIF4E)^38,39^ induce ERK/MAPK activation, resp. p38/MAPK pathways and COX enzyme formation (the COX-2 isoform is upregulated only in the presence of inflammation, COX-1 is constitutively expressed in tissues).

Prostaglandins (PGs) are prostanoids formed from arachidonic acid due cyclooxygenases action^22^, a family of lipoid hormones PGD2, PGE2, PGF2, and PGI2 that are involved in controlling reproduction (ovarian and menstrual cycle, fertility, and embryo implementation) and associated pathologies (menorrhea, dysmenorrhea, endometriosis)^37^. PGE2 in chronic inflammation induces inflammatory symptoms such as pain, edema, and phagocytosis^40^. PGE2 affects the stimulation of the angiogenic cytokine VEGF at the time of stromal implantation and decidualization, acts as a vasodilator, and thus promotes the growth of ectopic lesions^41^. MIF stimulates COX-2, thereby increasing the expression of PGE2 and key steroidogenic genes for local non-physiological overproduction of 17β-estradiol (E2) from cholesterol in endometriotic cells^42^.

PGE2-induced COX-2 activity is inhibited by E2 through p38/ERK/MAPK pathway signalling activity by binding to specific estrogen receptors ERα/β and affecting target genes^23,43,44^. PGE2 also regulates a number of metalloproteinases (MMPs—calcium-dependent zinc-containing endopeptidases), especially MMP-2/9, which are involved in the process of angiogenesis via intracellular MAPK pathways and VEGF^22^. MMPs are able to degrade collagen and elastin in the extracellular matrix and thus promote cell invasiveness^45^. In addition to estrogen, progesterone (P4), which affects transcription genes (e.g. Ihh, Ptch, Ekpb52), MMPs (MMP-2/9), cytokines (IL-6/8/1β, TNFα) and decidualization markers (IGFBP-1, prolactin)^24^. In the menstrual cycle, the level of MMPs changes periodically depending on the phase of the cycle (see Table 1)^46^.

**Table 1:** Activity of MMPs during reproductive cycle of women; minimal activity: +, moderate activity: ++/+++, strong activity: ++++^46^.

Menstrual cycle phases							
Proteins	Menstrual		Proliferative		Secretory		
	Early	Late	Early	Late	Early	Mid	Late
MMP-1	++++	++++	+	+	+	+	+
MMP-2	+++	+++	++	++	++	++	++
MMP-3	++++	++++	+	+	+	+	+
MMP-7	++++	++++	++++	++++	+	+	+
MMP-8	++++	++	+	+	+	+	+
MMP-9	++	++	+	+	+	+	+
MMP-10	++++	++++	++	++	+	++	++
MMP-11	++++	++++	++++	++++	+	+	+
MMP-12	++++	+	+	+	+	+	+
MMP-14	+++	+++	++	++	++	++	++
MMP-15	++	++	++	++	++	++	++
MMP-16	+	+	++	++	++	+	+
MMP-19	++	++	++	++	++	++	++
MMP-26	+	+	++	++	++++	++	+

### Zinc and its role in endometriosis

Zinc (in ionized form of Zn(II)) is an essential trace element which highly regulated homeostasis inhibits the effect of ROS, suppresses the activity of inflammatory cytokines, enzymes and adhesion molecules that lead to local inflammation^47–49^. Intracellular zinc signals act in an autocrine (mainly on liposomes, mitochondria and nucleus), paracrine and endocrine manner (zinc-secreting cells)^50^. Zinc activates MMPs that have a tumour suppressor effect and also inhibits the effect of ROS^51^. Oxidative stress in combination with zinc deficiency^52^ results in excessive activation of MMPs^45^ (since Zn(II) acts as inhibitor of MMPs and prevents substrate binding and degradation)^53^ which leads to development of pro-inflammatory reactions^54^ that promote endometriotic lesion expansion and endometrial cell penetration occurs^53,55^.

Due to zinc extensive effect on inflammatory pathways, Zn(II) compounds possess great potential in treatment of inflammation and cancer.

Non-genomic signalling pathways of zinc^50^, in a zinc waves, mediate the cell response together with calcium influx, activation of the ERK/MAPK pathway through specific CK2 (casein kinase 2)-mediated phosphorylation, as well as PI3K and mTOR pathways, and ZIP7 channel phosphorylation^48,49^. These signalling pathways lead to genomic (transcriptional) responses, e.g. MTF-1 regulates target genes with the MRE region (transcription factors, developmental and cell cycle regulating genes)^53,56,57^, or by chromatin modification with ZnF (C2H2-type Zinc finger proteins—transcription factors acting as tumour suppressors as well as oncogenes)^58–60^, or by regulating tumour cell migration and invasion^60^.

### Non-steroidal anti-inflammatory drugs (NSAIDs) as potentially suitable ligands

Most non-steroidal anti-inflammatory drugs (NSAIDs) are non-selective COX1-3 inhibitors^61^. The long-term use of NSAIDs has been shown to reduce the risk of cancer (breast, ovarian, prostate), induce apoptosis, inhibit proliferation, invasiveness, migration, and metastasis^62–65^.

The anti-inflammatory drugs also induce cell death through activation of Bax^66,67^, inhibition of prostaglandin H-synthase (COX), further affect glucose metabolism, regulate tumour suppressor gene expression for PTEN, and MAPK phosphatase 3, as well as intracellular Calcium (antagonist to Zinc)^68^. Hence, NSAIDs as well as their metal complexes are currently also investigated as potential anticancer agents.

We focused our present study on a member of fenamate NSAIDs—niflumic acid^63^. Niflumic acid (NFA/Hnif) is widely used in the treatment of painful menstruation^62^. The main role of NFA is induce changes in the intracellular level of Calcium^69–73^ the reduction of which leads to inactivation of the hyperosmotic induction of COX-2^74,75^. NFA at the concentration higher than that required for COX-2 inhibition leads to the accumulation of arachidonic acid, the increased concentration of which induces apoptosis by the involvement of caspase-3 and alter mitochondrial permeability^76^. The apoptotic effect of niflumic acid is manifested by down-regulation of the ERK/MAPK cascade^77^, reduction of MMP-2/9 activity^62^, and increases the apoptotic effect^78^.

In an attempt to develop multi-target drug for treatment of endometriosis and related endometrial cancers and based on our previous studies of metal(II) niflumates we have designed and studied [Zn(*neo*)(*nif*)_2_] complex which contains NSAID directly bonded to the zinc and might combine the positive effects of both components on endometriotic lesions.

## Material and methods

### Preparation and characterization of [Zn(neo)(nif)_2_]

Studied complex [Zn(*neo*)(*nif*)_2_] was prepared in crystalline according to the previously reported procedure^79^. The structure of the complex was verified by single crystal X-ray diffraction analysis as well as spectral methods (IR, UV–VIS, fluorescence). The solution of the complex for the experiments with hTERT and 12Z cell lines was prepared by dissolving pure crystals of the complex in DMSO and subsequent dilution of the stock solution to the desired concentration by used medium (DMSO concentration in final solutions was < 0.5%). Molecular structure model was created by using CCDC Mercury 2020.1 software.

### Cell lines 12Z and hTERT

Human telomerase reverse transcriptase (hTERT) epithelial cells and 12Z endometriosis epithelial cells (ExPASy 12Z) were cultured in Dulbecco's Modified Eagle's Medium (DMEM) with 25 mM glucose, 2 mM glutamine, 10% Fetal Bovine Serum (FBS), 100 U/ml of penicillin, 0.1 mg/ml streptomycin, and 1.25 g/ml amphotericin in a thermoincubator at 37 °C and 95% O_2_, 5% CO_2_ atmosphere.

Cell culture 12Z was a gift from prof. Anna Starzinski-Powitz (Goethe-Universität Frankfurt), acquired in accordance with ethical rules. The hTERT cell line combines the in vivo properties of primary cells with the in vitro viability (ATCC hTERT).

### xCELLigence assay

xCELLigence RTCA SP system (Roche Applied Science, Basel, Switzerland) measures changes in impedance (apparent resistance in terms of cell index) of built-in gold sensory microelectrodes at the bottom of E-plate 96 wells (ACEA Biosciences, Agilent, cat. no.: H029085). Measurements by xCELLigence assay on 12Z and hTERT cell lines were performed in biological triplicates, seeded at the density of 8000 cells/well of 12Z and 20,000 cells/well of hTERT (based on previous optimization of ideal culture conditions measurements—cell concentration titration measurements). Background was measured at 100 µl DMEM medium in an E-plate 96 in RTCA SP station (ACEA Biosciences) placed in the thermoincubator at 37 °C and 95% O_2_, 5% CO_2_ atmosphere. Subsequently, a suspension of 12Z cells and hTERT in a volume of 80 µl per well was added. After about 24 h of incubation, another 20 µl of DMEM medium with study substances was added to the medium with a final concentration of 100 µM/50 µM/10 µM/1 µM for *cis*-platin (*cis*Pt), niflumic acid, Zn complex, ZnCl_2_, neocuproine (100 μM), and finally 0.5% DMSO. The Cell Index (CI—the impedance of gold electrodes in the bottom of the 96-well plate dependent from a cell adhesion) was measured for 94 ± 2 h every 15 min. Each experimental group was measured in triplicate. The slope interval was generated using RTCA software (ACEA Biosciences), which was used to evaluate the CI ratio. This methodology demonstrated the cytotoxic effect of the studied complex, whose CI was normalized to the same value at the normalization time point.

### Cell viability: Trypan Blue Assay

The proliferative capacity and viability of 12Z and hTERT cell lines was detected using the Trypan Blue Staining Assay. Cells were seeded at the density of 150 000 per well in a 6-well plate and cultured for 72 h. Cells were affected with the 10 µM [Zn(*neo*)(*nif*)_2_] before starting cell proliferation measurements. The percentage of viable cells (at 24 h) was calculated.

### Gelatine zymography

The collected media supernatants of 12Z and hTERT cells were mixed with sample buffer under non-reducing conditions. The samples were fractionated in 10% polyacrylamide gels containing 0.15% gelatine with electrophoresis running at constant 120 V. After electrophoresis, the gels were washed with 2.5% Triton X-100 for 2 × 30 min in agitation and incubated in development buffer (10 mM CaCl_2_, 0.005 mM ZnCl_2_, 100 mM Tris–HCl pH 7.4) for 48 h at 37 °C. After incubation, the gels were stained with 0.5% Coomassie Brilliant Blue R-250 in the solution of 40% isopropanol and 10% acetic acid for 1 h at room temperature and distained for 4 h in the solution of 40% methanol and 10% acetic acid, and for 20 h in the solution of 5% methanol, 10% acetic acid. Gelatinolytic activities were defined as white bands on a blue background. Proteolytic activity for pro-MMP-9, active-MMP-9, pro-MMP-2, and active MMP-2 in the gel was visualized at 92, 82, 72, and 62 kDa, respectively. The zymogram of each sample was run in duplicate.

### Gene expression (q)RT-PCR

The total RNA was isolated with RNeasy Micro Kit (Qiagen, Hilden, Germany) from in the cool PBS harvested cells hTERT and 12Z (non-treated, treated with 10 µM [Zn(*neo*)(*nif*)_2_], and with 10 µM *cis*Pt for 24 h). The sample concentration and purity was analysed by Nanodrop 2000c (Thermofisher Scientific, Waltham, MA, USA). The RNA samples were transcribed into cDNA by ProtoScript First Strand cDNA Synthesis Kit (New England BioLabs, Ipswich, MA, USA). The real-time PCR was provided using SensiMIX SYBR No-ROX (Bioline, London, UK) and forward/reverse primer of *Gapdh* (Invitrogen, Carlsbad, CA, USA), *Mmp*-2 (Invitrogen, Carlsbad, CA, USA), *Mmp*-9 (Invitrogen, Carlsbad, CA, USA) on Rotor Gene Q.

### Spectral measurements of cell nuclei

The nuclei of unaffected cells as well as cells treated with [Zn(*neo*)(*nif*)_2_] (10 μM concentration) were separated from the cell suspension by ultracentrifugation using standard sucrose density gradient method and lysed by dilution of the resulting suspension with distilled water (1:7). The simultaneous measurement of absorption and fluorescence spectra of the samples were performed on Horiba Dual-FL CCD spectrofluorometer at room temperature. The obtained data were analysed with Origin Pro 8 (v8.0891).

### Statistical analysis

Cell index (CI) and normalized Cell Index (nCI) for real-time dynamic cytotoxicity assessment (N = 3) and slope calculations for the migration assessments were calculated automatically by the RTCA Software of the RTCA system. Numerical data were expressed as a mean ± standard deviation.

The final mRNA level of *Mmp*-2, and *Mmp*-9 were evaluated by comparative quantification and ΔΔCt values using the Rotor Gene Q Software (Qiagen, Hilden, Germany) and obtain the relative amount of target mRNA determined by the formula: target gene1 = 2^−ΔΔCt^ of target gene1.

Statistical differences between the means for the different groups were evaluated with GraphPad Prism 5.0 (GraphPad software, La Jolla, CA, USA) using the Multiple *t* test with the level of significance and the One-way ANOVA with *p* values: *p* < 0.05, *p* < 0.01, *p* < 0.001 in analysis of cytotoxicity measurements.

## Results and discussion

**Figure 2:**
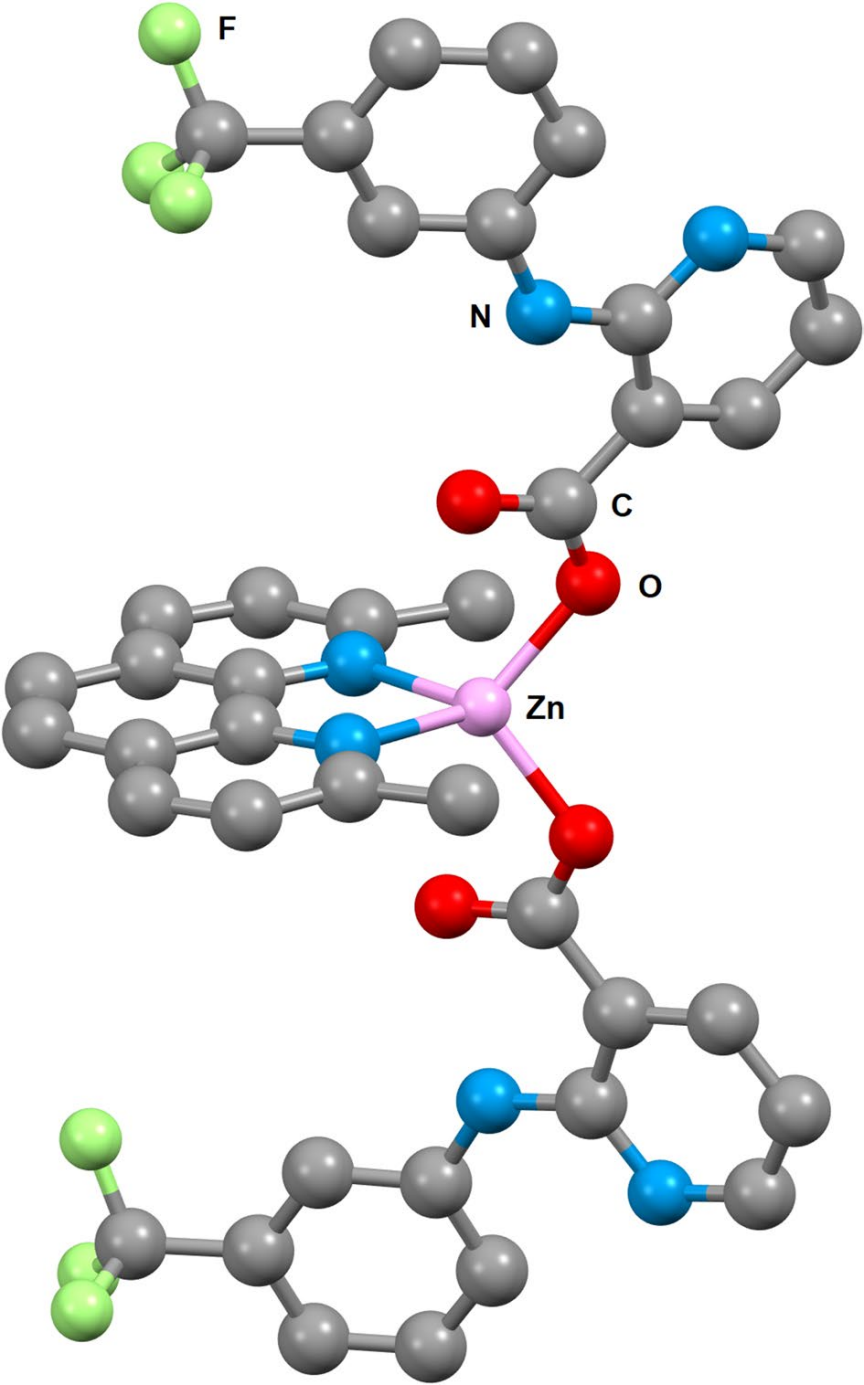
View of the molecular structure of studied complex [Zn(*neo*)(*nif*)_2_]. Hydrogen atoms were omitted for the sake of clarity (https://www.ccdc.cam.ac.uk/solutions/csd-core/components/mercury/).

The recently reported complex [Zn(*neo*)(*nif*)_2_] has shown promising potential in the preliminary cytotoxicity studies and thus it was selected for a more thorough investigation. Within the structure of the complex, central Zn(II) atoms are tetracoordinated by two oxygen atoms of niflumato ligands bonded in monodentate manner and two nitrogen atoms of chelate bonded neocuproine ligand (Fig. 2). Whereas the niflumato ligands are presumably the biologically most active part of the complex, the aromatic neocuproine ligand provides the additional stability and enhances its DNA as well as protein binding properties. The molecular structure of the complex resembles binding domains of C2H2 coordinated ZnF.

Our previous measurements of cytotoxicity interpreted as IC50 values show (Table 2) that the studied complex has approximately 4.6-fold greater cytotoxic effect for rapidly proliferating 12Z cells compared to hTERT cells^79^. The *cis*-platin (*cis*Pt) was selected as comparative compound to detect the efficiency of Zn-Nif complex.

**Table 2:** IC_50_ calculated from CI at a time point (72 h) versus concentration 79.

	IC_50_ 12Z (mmol/l)	IC_50_ hTERT (mmol/l)
cisPt	>0.250 (0.333)	>0.250 (1.181)
[Zn(*neo*)(*nif*)_2_]	0.000895	0.00412
MIX (ZnCl_2_+H*nif*+*neo*)	>0.100 (0.225)	>0.100 (0.899)

The values of IC5_0_ of the complex ([Zn(*neo*)(*nif*)_2_]), cis-platin (*cis*Pt), and the mixture of the complex components (MIX=ZnCl_2_+H*nif*+*neo*).

*Values in the brackets represent calculated IC_50_ values based on extrapolation.

### Identification of acute and long-term response upon [Zn(neo)(nif)_2_] treatment

**Figure 3:**
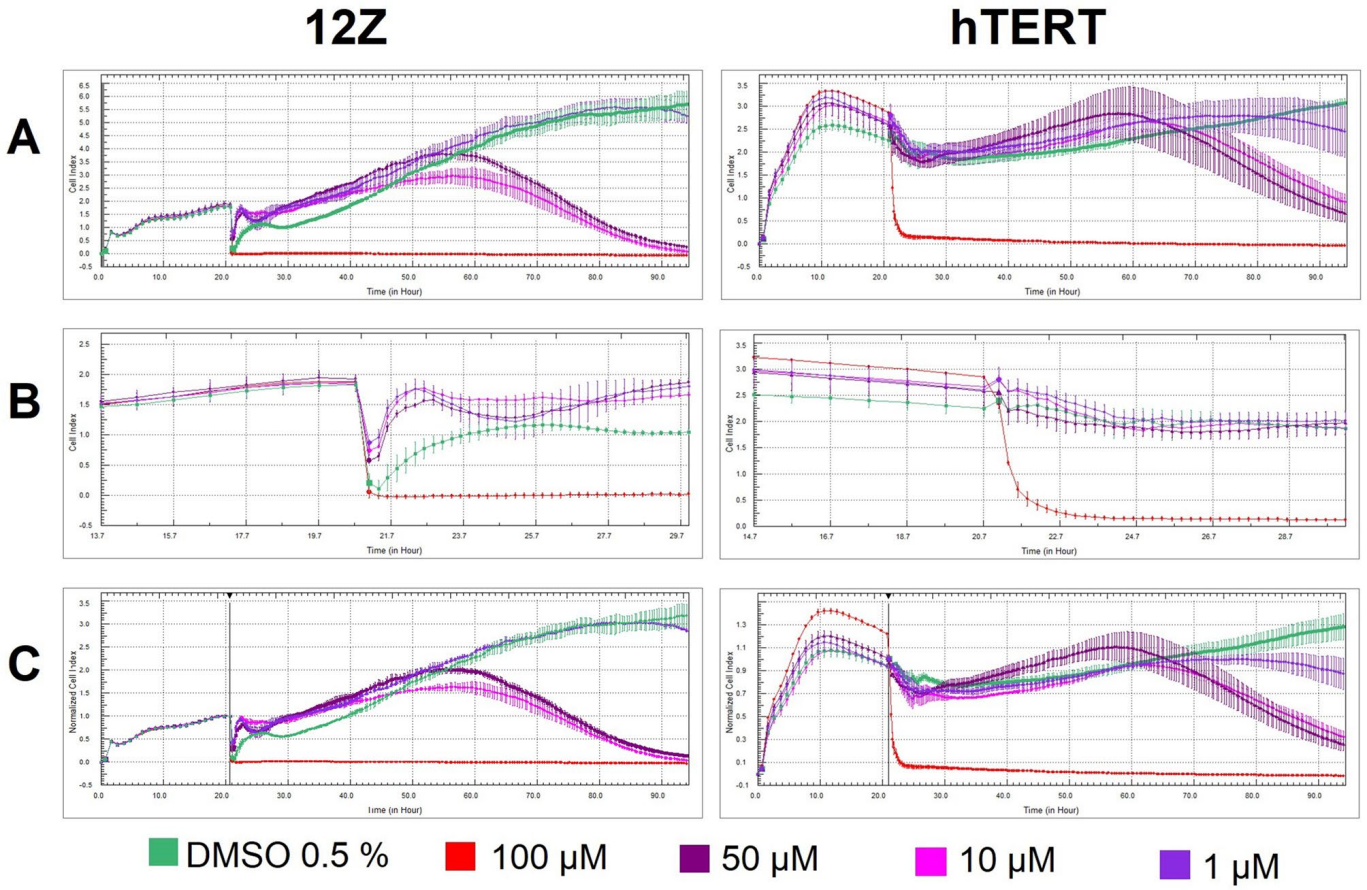
Cell Index (CI) and Normalized Cell Index (nCI) for 12Z and hTERT cells affected with [Zn(*neo*)(*nif*)_2_] (experimental cells) and 0.5% DMSO (control cells). Data from xCELLigence RTCA SP system. (**A**) Cell Index raw data; (**B**) Detail of short time period prior to and following the addition of complex; (**C**) Normalized Cell Index raw data.

We monitored the response profile of endometrial cell lines hTERT and 12Z at [Zn(*neo*)(*nif*)_2_] at concentrations of 100, 50, 10 and 1 μM using the xCELLigence Assay. Time-lapse data (approximately for 93 h) describe in Fig. 3A the rapid and transient reduction in cell adhesion induced by the 100 µM complex in both cell lines (hTERT and 12Z). The response of the cells to the Zn-Nif at a concentration of 50 and 10 µM had a long-term effect. 12Z cells responded after 24–30 h, whereas hTERT cells did not respond until 40–48 h.

Figure 3B is a visualization of a short section before and after the studied Zn-Nif. It is clear from the graph that the response of the cells of the endometriotic line 12Z to the added [Zn(*neo*)(*nif*)_2_] is prompt and at the concentration of 100 µM is fatal response. An initial increase in cell index (CI) was observed in 12Z cells which is thought to correspond to an increase in inflammatory pathways in the cells and a subsequent decrease in CI is followed by a gradual progression of cell death in the long-term phase after treatment with Zn-Nif at 50 and 10 µM. In contrast, we did not observe a similar increase in CI in hTERT cells immediately after the addition of the complex. The response of both cell lines to the 1 μM concentration of Zn-Nif correlated with the behaviour of the control unaffected cells. Figure 3C shows the nCI at the time of addition of [Zn(*neo*)(*nif*)_2_]. A direct reduction in adhesion of 12Z cells by 65–75% is visible compared to the hTERT cell line were no significant change in impedance was observed within 15 min from the addition of complex to the cells (except for the concentration of 100 µM, where in both immediate lethal response was observed in the cell lines).

The nCI values depending on the concentration and time of action of the complex show that the concentration of 10 µM has the expected cytotoxic effect on cells. This cytotoxic effect was more significant for cell line 12Z than for hTERT which is also indicated by Doubling time (in the total time of action of the Zn-Nif) for cell line 12Z has value − 28.5 ± 2.3 and for hTERT line has value − 141.8 ± 20.9. The slope (in the total time of action of the Zn-Nif) has value − 0.03 ± 0.003 for 12Z cells and − 0.003 ± 0.0004 for hTERT cells.

These data indicate that hTERT cells respond significantly more slowly and less destructively to the studied complex than rapidly proliferating 12Z cells. Apparently, the highest tested concentration of the complex (100 μM) induces cell death in both lines after substance addition immediately. However, the complex concentration of 50 and 10 μM leads to a long-term response in the form of a continuous decrease in CI with possibly an apoptotic end. On the other hand, this effect was not observed for the lowest tested concentration of 1 µM. The measured data using the xCELLigence Assay were also confirmed by another terminal method—Trypan Blue Assay.

**Figure 4:**
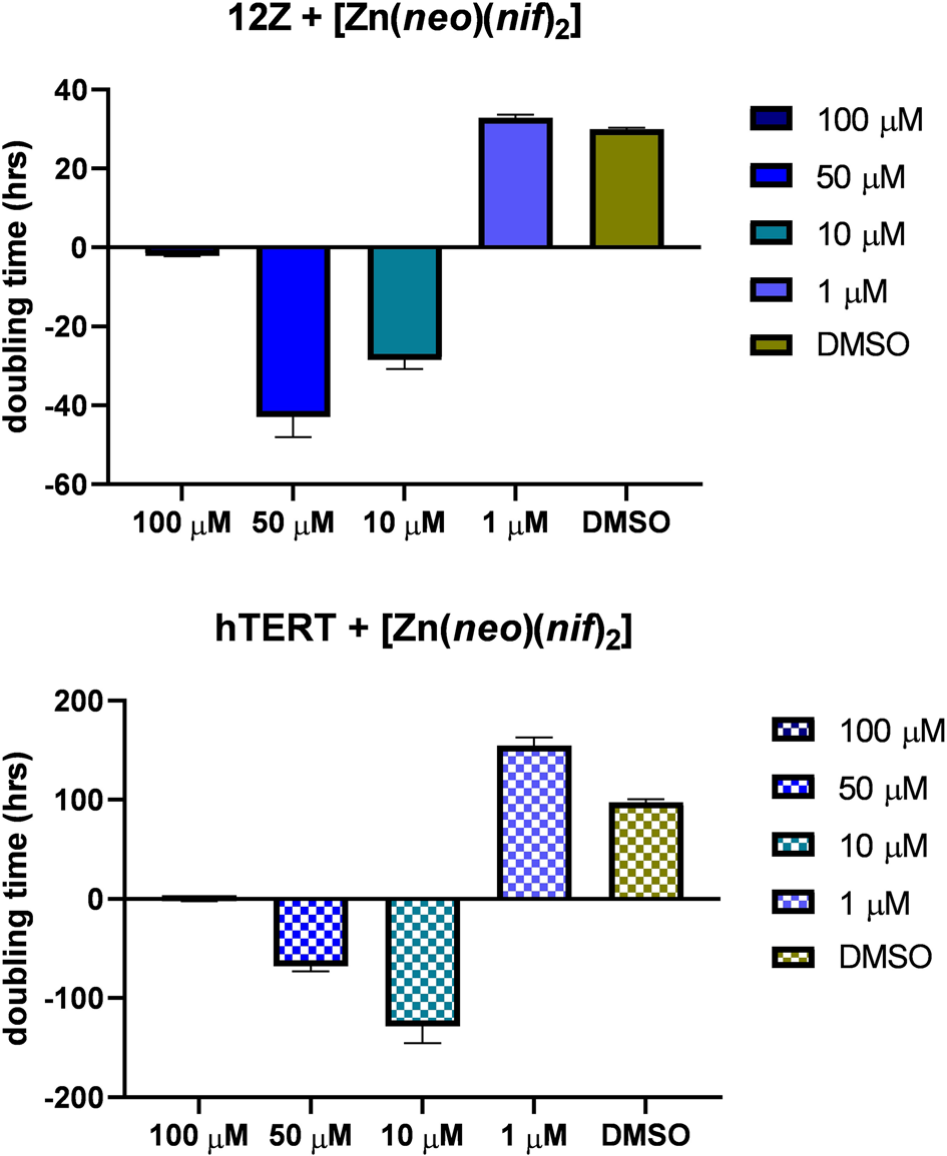
xCELLigence data for Doubling of 12Z and hTERT cells treated with [Zn(*neo*)(*nif*)_2_] in different concentration.

Cell adhesion (slope in the time interval 0–12 h) of affected cells with 10 µM complex was for cells 12Z 4-times lower than for hTERT cells. Proliferation of monitored cells (slope in the time interval 12–24 h) of affected cells 12Z 10-times lower than in control cells. Survival of affected cells (slope in the time interval 24–48 h) for 12Z 2-times higher than for hTERT. Cell death (slope in the time interval 48–72 h) for 12Z 5-times higher than for hTERT cells. The values of doubling time (Fig. 4) and slope values for adhesion, proliferation, survival, or cell death for individual time windows are in supplementary ST 1 (significance values in ST 2 and ST 3).

The inhibition of 12Z cell proliferation 12 h after treatment with 10 μM was 87.64%. Proliferation of hTERT cells 12 h after treatment with the complex (10 μM) was unaffected and represented 116.25%.

### Effect of the [Zn(neo)(nif)_2_] on cell nuclei

**Figure 5:**
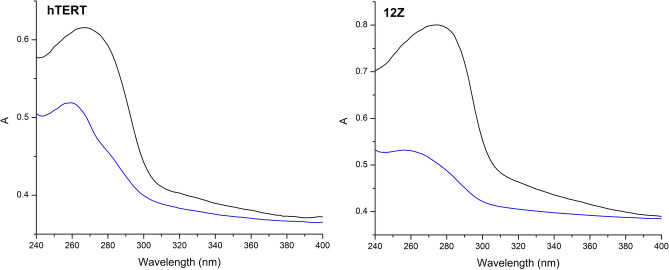
Absorption spectra of cell nuclei samples from unaffected cells (black) and cells treated with complex (10 μM) for 24 h (blue) for hTERT (left) and 12Z (right) cell lines.

The potential effects of the complex on nuclei and possible interaction of the complex with nuclear genomic DNA were explored by absorption and fluorescence measurements. The comparison of the absorption spectra of the lysed cell nuclei samples from the unaffected cells and cells treated with [Zn(*neo*)(*nif*)_2_] (10 μM) for 24 h has revealed a visible decrease of the intensity of broad nucleoprotein absorption band in the samples isolated from the treated cells of both hTERT and 12Z cell lines (Fig. 5). To evaluate observed differences between the samples, ratio between absorbance of samples from treated cells (A) and samples from unaffected cells (A0) at 260 and 280 nm wavelengths were calculated (Table 3). The absorbance at selected wavelengths correspond to the absorption of the nucleic acids (260 nm) and nuclear proteins (280 nm), respectively, and therefore can be used to determine the changes in the structure of these two nucleoprotein components. The hypochromic effect observed in samples from the cell treated with complex clearly indicate structural changes of the nucleic acids which are visibly more pronounced in case of 12Z cell line. Decrease in absorbance at 260 nm might be associated with both direct interaction of the complex with nucleic acids which was confirmed by our previous studies on isolated DNA samples^79^, as well as by affecting transcription factors^80^. Obtained results are also in line with the higher cytotoxicity of the complex against 12Z cell line which is reflected by more significant hypochromic effect of the absorbance in lysed cell nuclei sample isolated from the affected cells. In addition to the absorption measurements, a full excitation-emission matrix fluorescence measurements were carried out, however due to the similar position of fluorescence maxima of studied complex and nucleic acids it was not possible to confirm the presence of the complex within the isolated nuclei samples by this method.

**Table 3:** Selected parameters calculated from absorption spectra of cell nuclei samples from hTERT and 12Z cell lines (A0=absorbance of samples from unaffected cells; A=absorbance of samples from cells treated with complex).

Parameter	hTERT	12Z
A(260)/A_0_(260)	0.851	0.685
A(280)/A_0_(280)	0.769	0.615
A_0_(260)/A_0_(280)	1.03	0.98
A(260)/A(280)	1.29	1.09

### Effect of the [Zn(neo)(nif)_2_] on the cell viability

Cell viability Trypan Blue test showed inhibition of cell proliferation for endometriotic cells 12Z and healthy control hTERT cells after [Zn(*neo*)(*nif*)_2_] addition in concentration of 10 µM. Our present study identified Zn-Nif as possible effector of MAPK and COX-2 signalling, inhibition of which could induce apoptosis or apoptosis-like cell death.

**Figure 6:**
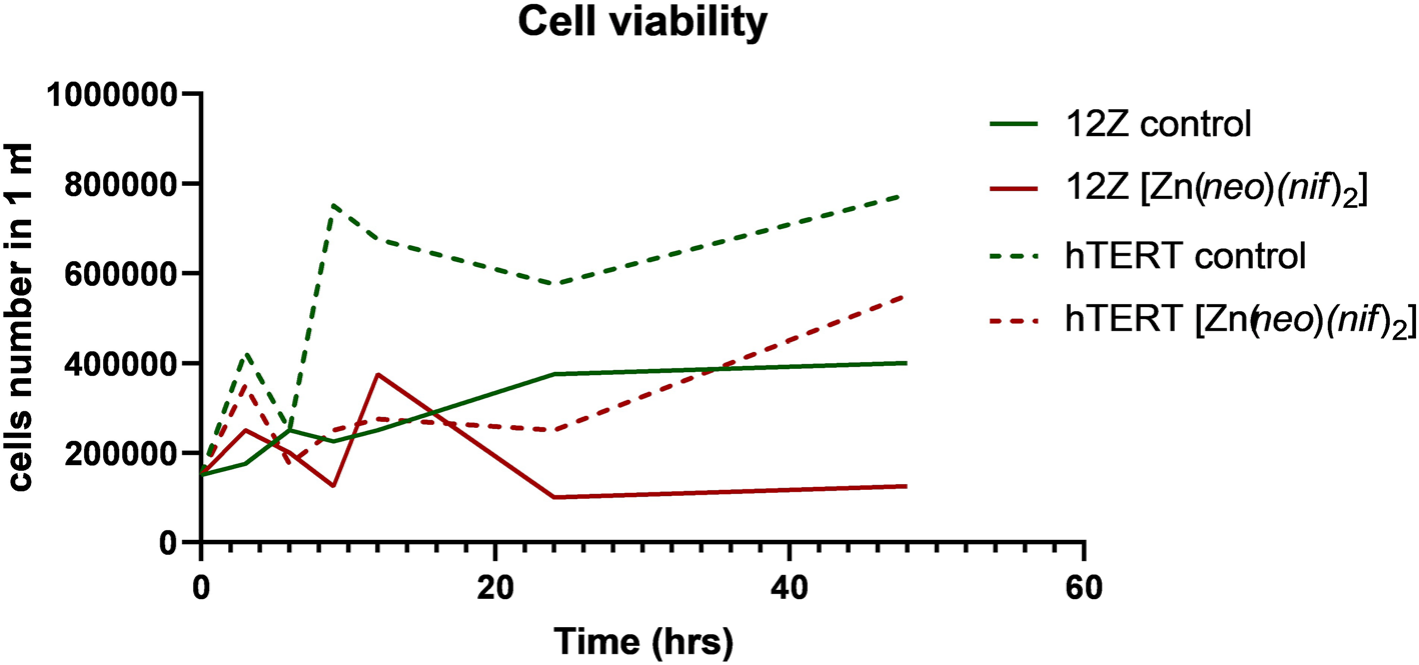
Cell viability of non-endometriotic hTERT cells and endometriotic 12Z cells with and without Zn-Nif complex.

We observed that 12Z cells affected by [Zn(*neo*)(*nif*)_2_] had about 69% decrease of live cells at 48 h and hTERT cells which had about 29% decrease of live cells at 48 h (Fig. 6).

The cytotoxic, anti-proliferative, and inhibitory effect was significantly greater in endometriotic cell line 12Z than in the control endometrial cell line hTERT as already declared on the basis of the results obtained by the xCELLigence Assay and cell viability Trypan Blue Assay. The activity of the Zn-Nif complex might be a result of its multi-target approach (anti-inflammatory, anti-cancer, pain suppression, activation of a tumour suppressor MMPs, and inhibition of the ROS effect)^51,76,78^. Based on results of xCELLigence RTCA analysis we have found that while the concentration of 1 μM Zn-Nif has no cytotoxic effect in any interval of action on studied cell lines from, the concentration of 10 μM has the desired cytotoxic effect on the treated 12Z cell culture but not on the treated hTERT cells after 24 h. In our study, we observed that the Zn-Nif inhibits proliferation, migration, and showed a selective cytotoxic effect which was manifested in a significant reduction of viable cells in 48 h by 69% in the 12Z cell line and by 29% in the hTERT cell line, respectively. These measurements show that hTERT cells are more resistant to the effect of [Zn(*neo*)(*nif*)_2_] compared to 12Z cells and also correlate with the theory that NSAIDs complexed with bio-metal are more effectively reduce proliferation than NSAID alone^79^.

### Effect of the [Zn(neo)(nif)_2_] on the gene expression

**Figure 7:**
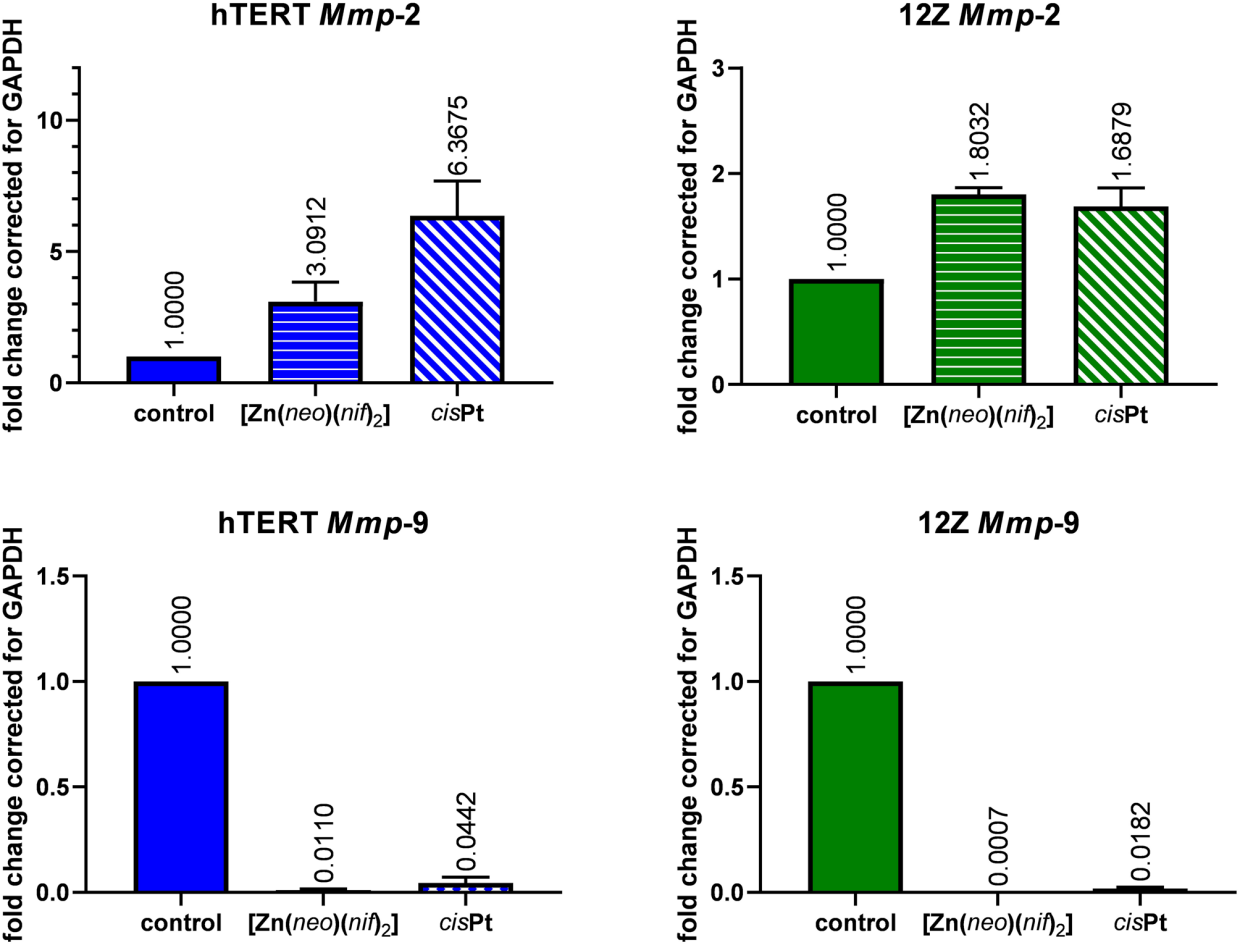
The relative gene expression of Mmp-2/9 in control (untreated cells hTERT or 12Z), cells treated with 10 µM [Zn(*neo*)(*nif*)_2_] or 10 µM cisPt for 24 h.

A significant change in gene expression of *Mmp*-2 and *Mmp*-9 (fold change to Gapdh) in the cells treated with 10 µM [[Zn(*neo*)(*nif*)_2_] was detected by RT-PCR and qRT-PCR. Relative target gene expressions as fold change are shown in the Fig. 7.

The non-endometriotic hTERT cells showed significant increase of relative *Mmp*-2 expression after 24 h incubation with 10 µM [Zn(*neo*)(*nif*)_2_] (*p* = 0.0458) and 10 µM *cis*Pt (*p* = 0.0005), and significant increase of relative *Mmp*-2 expression after 24 h incubation with 10 µM [Zn(*neo*)(*nif*)_2_] (*p* = 0.0002), and 10 µM *cis*Pt (*p* = 0.0004) in endometriotic 12Z cell line. In opposite, we analysed the significant decrease in hTERT of relative *Mmp*-9 expression after 24 h incubation with 10 µM [Zn(*neo*)(*nif*)_2_] (*p* < 0.0001) and 10 µM *cis*Pt (*p* < 0.0001), and the decrease of relative *Mmp*-9 expression after 24 h incubation with 10 µM [Zn(*neo*)(*nif*)_2_] (*p* < 0.0001), and with 10 µM *cis*Pt (*p* < 0.0001) in 12Z cells.

### Effect of the [Zn(neo)(nif)_2_] on active and latent form of MMPs

Following up on what was found by gene expression, we also performed the determination of MMP-2/9 by gelatin zymography as it is more sensitive than WB to these exopeptidases.

**Figure 8:**
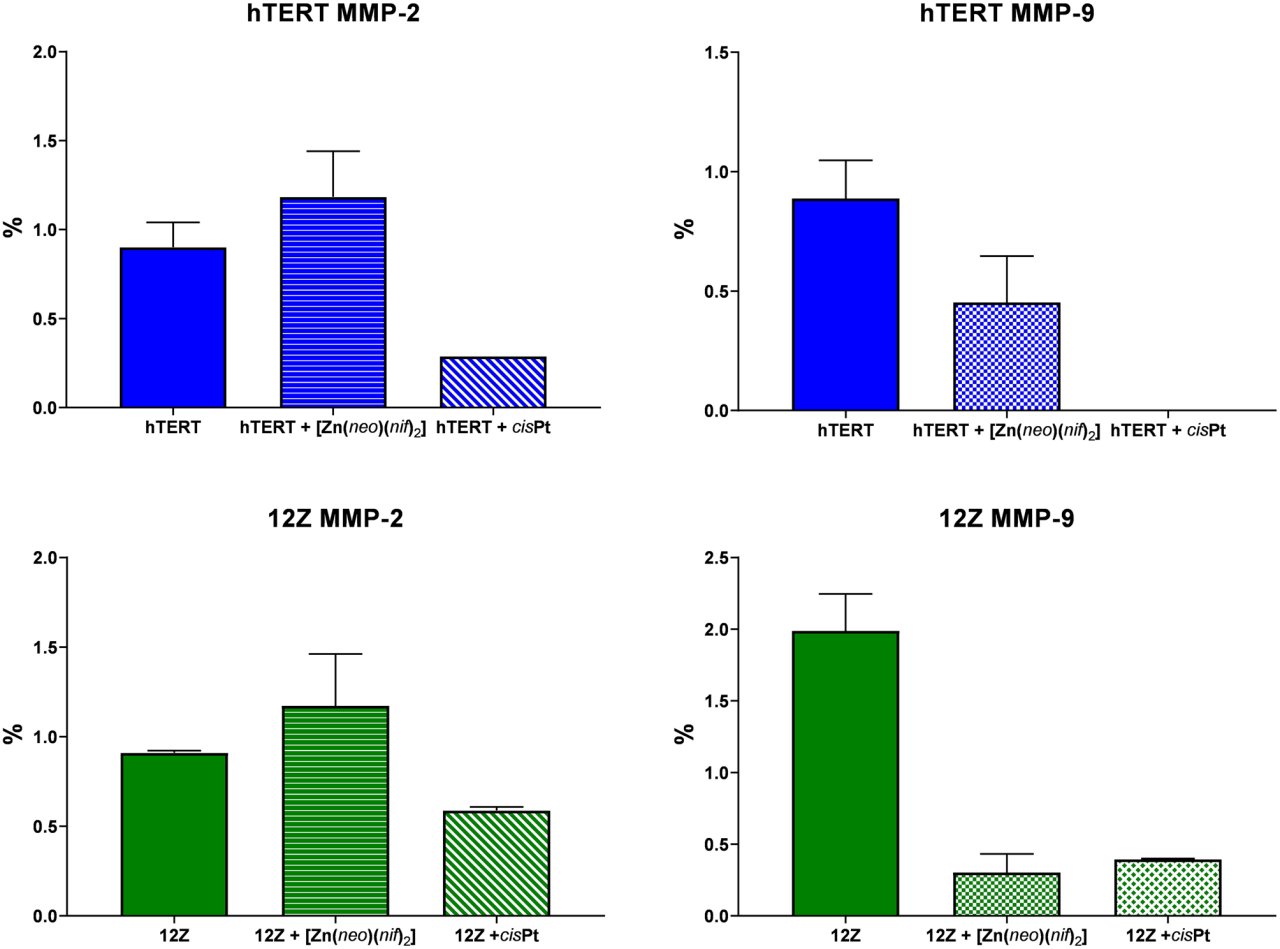
Total MMP-2/9 protein activity determined by gelatine Zymography in control (untreated cells hTERT or 12Z), cells treated with 10 µM [Zn(*neo*)(*nif*)_2_] or 10 µM cisPt for 24 h.

As shown by gelatine zymography (Fig. 8), treatment with *cis*Pt and Zn-Nif efficiently inactivated the release of proMMP-9 (latent MMP-9) into the medium of 12Z and hTERT cells. Latent MMP-9 in hTERT cell line treated with 10 µM [Zn(*neo*)(*nif*)_2_] dropped by 65%, and treated with 10 µM *cis*Pt was vanished.

ProMMP-9 in untreated 12Z cell line was increased twice compared to untreated hTERT cell line, 12Z cells treated with 10 µM [Zn(*neo*)(*nif*)_2_] decreased by 85% compared to untreated 12Z cells and ± 70% compared to untreated hTERT, and 12Z cells treated with 10 µM *cis*Pt decreased 80% compared to untreated 12Z and decreased 60% compared to untreated hTERT cells.

Latent MMP-2 in hTERT cell line treated with 10 µM [Zn(*neo*)(*nif*)_2_] increased by ± 25% and treated with 10 µM *cis*Pt was decreased about ± 70%. ProMMP-2 in untreated 12Z cell line was decreased by ± 12% compared to untreated hTERT cell line, 12Z cells treated with 10 µM [Zn(*neo*)(*nif*)_2_] increased by ± 25% compared to untreated 12Z cells, and ± 30% compared to untreated hTERT. Experimental group of 12Z cells treated with 10 µM *cis*Pt decreased ± 40% compared to untreated 12Z and ± 32% compared to untreated hTERT cells.

We hypothesize that this mechanism of action is induced by the regulation of responses to the pro-inflammatory agents MAPK pathways (ERK, p38 and JNK) in eutopic epithelial cells of endometriotic lesions. The effect of [Zn(*neo*)(*nif*)_2_] is on the transcriptional level in probably increased activity of TNFα, IL-1β, TGF-β, VEGF, and other regulatory genes (e.g. Zn-dependent genes) in endometriosis and at the same time affects the activity of MMPs (Zn inactivates pathological activation of MMPs and promotes tumour suppressor activity of MMPs)^51,74,78^. Elevated level of MMP-2/9 may cause with ectopic endometriosis advanced progression or at its proliferative stage. The [Zn(*neo*)(*nif*)_2_] effectively decreased MMP-9 which seems to have huge effect on proliferation of endometriotic cells. In opposite, we determined increased the MMP-2 protein levels after Zn(II) complex treatment (correlation with relative gene expression). The MMP-2 can have also protective function^81^ which seems to have an important role in our experiment and could explain the increased protein activity compared to decreased activity after *cis*Pt treatment.

## Conclusions

Our work demonstrates that the Zn-Nif is presumably affecting the activity of genes controlling the induction of apoptosis and may induce changes in the signalling mechanisms of inflammatory responses which can lead to cell cycle arrest. It is known that genes regulating inflammatory processes are influenced by the activity of zinc-finger proteins, the role of which can probably be simulated by Zn-complexes like [Zn(*neo*)(*nif*)_2_]. Therefore, we see great potential in further study of similar NSAID metal complexes.

Our results also suggest that xCELLigence assay could be a helpful tool for the future study of NSAID complexes with biometals and their application in the treatment of chronic inflammatory diseases such as endometriosis. Real-time analysis of the cytotoxic effect of experimental drugs is more appropriate than the end-point analysis in monitoring of the cells response to the studied substance and capturing significant metabolic changes^82^ and can contribute to finding the appropriate concentration of newly designed drugs such as [Zn(*neo*)(*nif*)_2_].

## Supplementary Information

Supplementary Information.
