# Supplementary material for: Zinc(II) niflumato complex effects on MMP activity and gene expression in human endometrial cell lines
Source: Sci Rep. 2021 Sep 27;11:19086. doi: 10.1038/s41598-021-98512-9 (PMC8476601; doi:10.1038/s41598-021-98512-9)
Supplement: Supplementary file 1 — Supplementary Information. [file 41598_2021_98512_MOESM1_ESM.docx]

**SUPPLEMENTARY**

**Zinc(II) niflumato complex: Effects on MMP activity and gene expression in human endometrial cell lines**

Miroslava Rabajdová^a^, Ivana Špaková^a^, Zuzana Klepcová^a^, Lukáš Smolko^a^, Michaela Abrahamovská^a^, Peter Urdzík^b^, Mária Mareková^a^

^a^ Department of Medical and Clinical Biochemistry, Pavol Jozef Šafárik University in Košice, Faculty of Medicine

^b^ Department of Gynaecology and Obstetrics, Pavol Jozef Šafárik University in Košice, Faculty of Medicine

^*^ corresponding author: jana.maslankova@upjs.sk, Department of Medical and Clinical Biochemistry, Pavol Jozef Šafárik University in Košice, Trieda SNP 1, 040 11 Košice, Slovakia

ST 1. xCELLigence RTCA SP system data of doubling time and slope for 12Z and hTERT cells affected with [Zn(*neo*)(*nif*)_2_] (experimental cells) and 0.5 % DMSO (control cells). Time range 0-24 hrs represents adhesion and spreading of cells, 24-48 hrs for proliferation phase of cells, 48-72 hrs for cell death or surviving phase after [Zn(*neo*)(*nif*)_2_] addition.

| **cell type + compound concentration** | **time (hrs)** | **[Zn(*neo*)(*nif*)_2_]** | | | |
| --- | --- | --- | --- | --- | --- |
|  |  | **doubling** | | **slope** | |
|  |  | SEM | SD | SEM | SD |
| 12Z (100 µM) | 0-24 | -17.6897 | 3.79 | -0.0071 | 0.0014 |
|  | 24-48 | -0.9812 | 0.1099 | -0.0098 | 0.0001 |
|  | 48-72 |  |  | -0.0076 | 0.0001 |
|  | | | | | |
| 12Z (50 µM) | 0-24 | 15.1188 | 0.5830 | 0.1598 | 0.0029 |
|  | 24-48 | -105.0572 | 21.5005 | -0.0379 | 0.0067 |
|  | 48-72 | -7.1348 | 0.0650 | -0.1882 | 0.0037 |
|  | | | | | |
| 12Z (10 µM) | 0-24 | 24.0142 | 1.53 | 0.0746 | 0.0026 |
|  | 24-48 | -140.3427 | 30.4217 | -0.0178 | 0.0033 |
|  | 48-72 | -5.4966 | 0.1059 | -0.1301 | 0.0023 |
|  | | | | | |
| 12Z (1 µM) | 24-48 | 18.9161 | 0.7108 | 0.0838 | 0.0024 |
|  | 48-72 | 31.2992 | 0.5543 | 0.1045 | 0.0012 |
|  | 48-72 | 217.4784 | 26.0436 | 0.0200 | 0.0021 |
|  | | | | | |
| hTERT (100 µM) | 0-24 | -9.2980 | 0.6435 | -0.0073 | 0.0015 |
|  | 24-48 | -1.5028 | 0.1966 | -0.0009 | 0.0000 |
|  | 48-72 |  |  | -0.0005 | 0.0000 |
|  | | | | | |
| hTERT (50 µM) | 0-24 | 70.3457 | 6.86 | 0.0082 | 0.0007 |
|  | 24-48 | -494.0069 | 577.3598 | -0.0014 | 0.0008 |
|  | 48-72 | -13.3698 | 0.1209 | -0.0279 | 0.0002 |
|  | | | | | |
| hTERT (10 µM) | 0-24 | -271.1038 | 216.4130 | -0.0022 | 0.0009 |
|  | 24-48 | 77.0059 | 4.93 | 0.0077 | 0.0005 |
|  | 48-72 | -16.7909 | 0.1989 | -0.0240 | 0.0001 |
|  | | | | | |
| hTERT (1 µM) | 0-24 | -342.5254 | 326.2892 | -0.0019 | 0.0008 |
|  | 24-48 | 70.5480 | 1.26 | 0.0088 | 0.0001 |
|  | 48-72 | -151.4879 | 9.38 | -0.0043 | 0.0002 |
|  | | | | | |
| 12Z (DMSO 0.5 %) | 0-24 | 10.1448 | 0.9993 | -0.0033 | 0.0006 |
|  | 24-48 | 25.2627 | 0.4133 | 0.0098 | 0.0001 |
|  | 48-72 | 114.9706 | 2.33 | 0.0102 | 0.0001 |
|  | | | | | |
| hTERT (DMSO 0.5 %) | 0-24 | -183.3611 | 41.0264 | 0.6823 | 0.0223 |
|  | 24-48 | 65.6853 | 0.4535 | 0.9417 | 0.0083 |
|  | 48-72 | 78.3449 | 0.5447 | 0.3001 | 0.0068 |

ST 2 – Doubling and Slope P value compared to DMSO control

| **One-way ANOVA Sidak’s multiple comparisons test** | | | | |
| --- | --- | --- | --- | --- |
|  | **100 µM** | **50 µM** | **10 µM** | **1 µM** |
| **doubling** | | | | |
| **12Z + [Zn(*neo*)(*nif*)_2_]** | <0.0001 | <0.0001 | <0.0001 | ns |
| **hTERT + [Zn(*neo*)(*nif*)_2_]** | <0.0001 | <0.0001 | <0.0001 | <0.0001 |
| **slope** | | | | |
| **12Z + [Zn(*neo*)(*nif*)_2_]** | <0.0001 | <0.0001 | <0.0001 | <0.0001 |
| **hTERT + [Zn(*neo*)(*nif*)_2_]** | <0.0001 | <0.0001 | <0.0001 | <0.0001 |

**ST 3** – Doubling and Slope P values compared to DMSO control in time intervals (0-24, 24-48, 48-72 hrs)

| **Multiple t-tests** | | | | |
| --- | --- | --- | --- | --- |
|  | **100 µM** | **50 µM** | **10 µM** | **1 µM** |
| **doubling** | | | | |
| **12Z + [Zn(*neo*)(*nif*)_2_]** | | | | |
| 0-24 | 0.0003 | 0.0017 | 0.0002 | 0.0002 |
| 24-48 | <0.0001 | 0.0005 | 0.0007 | 0.0001 |
| 48-72 | - | <0.0001 | <0.0001 | 0.0025 |
| **hTERT + [Zn(*neo*)(*nif*)_2_]** | | | | |
| 0-24 | 0.0018 | 0.0005 | 0.5282 | 0.449 |
| 24-48 | <0.0001 | 0.1684 | 0.0167 | 0.0033 |
| 48-72 | - | <0.0001 | <0.0001 | <0.0001 |
| **slope** | | | | |
| **12Z + [Zn(*neo*)(*nif*)_2_]** | | | | |
| 0-24 | <0.0001 | <0.0001 | <0.0001 | <0.0001 |
| 24-48 | <0.0001 | <0.0001 | <0.0001 | <0.0001 |
| 48-72 | <0.0001 | <0.0001 | <0.0001 | <0.0001 |
| **hTERT + [Zn(*neo*)(*nif*)_2_]** | | | | |
| 0-24 | 0.0128 | <0.0001 | 0.1530 | 0.0724 |
| 24-48 | <0.0001 | <0.0001 | 0.0020 | 0.0003 |
| 48-72 | <0.0001 | <0.0001 | <0.0001 | <0.0001 |
